# Effects of Baduanjin Exercise on Antihypertensive Medication Reduction in Older Patients with Hypertension: A Study Protocol for a Randomized Controlled Trial

**DOI:** 10.1155/2021/8663022

**Published:** 2021-07-22

**Authors:** Ran Zhao, Shengjie Yang, Dan Li, Longtao Liu, Yanwei Xing, Min Wu

**Affiliations:** ^1^Guang'anmen Hospital, China Academy of Chinese Medical Sciences, Beijing, China; ^2^Beijing University of Chinese Medicine, Beijing, China; ^3^Xiyuan Hospital, China Academy of Chinese Medical Sciences, Beijing, China

## Abstract

**Background:**

Hypertension is a serious global public health problem, and its incidence increases with age. Research has suggested that multiple antihypertensive prescriptions may be harmful to older individuals with hypertension. Baduanjin, a widely practiced physical activity in China, has been used as an adjuvant therapy to antihypertensive treatment in older individuals with hypertension. Therefore, the aim of the current study is to investigate whether Baduanjin exercise could reduce the need for antihypertensive medications in older patients.

**Methods:**

This single-center, open, randomized controlled trial will be conducted in China. Seventy eligible participants will be randomly assigned to either the 12-week Baduanjin exercise group or the 12-week aerobic exercise group (5 times a week), at a ratio of 1 : 1. The primary outcome will include the proportion of participants who reduce their antihypertensive medications. The secondary outcomes will include the changes in mean office systolic and diastolic pressure, 24-h ambulatory blood pressure, health-related quality of life (based on SF-36), and gut microbiota composition. *Discussion.* The results of this study will provide powerful evidence for the use of Baduanjin as an adjuvant therapy for antihypertensive medication reduction in older people with hypertension and therefore find the best treatment for hypertension. This trial is registered in the Chinese Clinical Trials Registry (ChiCTR1900024429).

## 1. Introduction

Hypertension (HTN) is an age-related chronic disease, and its incidence increases with age [1]. According to a survey from 2012 to 2015, the prevalence of HTN among elderly individuals aged ≥65 years is 55% in China [2]. Patients with HTN are at higher risks of cardiovascular diseases and stroke, with annual deaths reported to be up to 1 million [3]. HTN has significant social and economic costs, and its management is of high importance.

Angiotensin-converting enzyme inhibitors, angiotensin II receptor blockers, beta-blockers, calcium channel blockers, and diuretics are recommended for HTN by the European Society of Cardiology (ESC) and the European Society of Hypertension (ESH) [4]. In China, more than 35% of patients with HTN aged ≥65 years control their blood pressure (BP) by taking two or more antihypertensive medications as prescribed [5]. Research has suggested that multiple antihypertensive prescriptions may be harmful to older individuals [6, 7]. Indeed, it has been clinically reported that a considerable reduction in BP increases the risk of hypotension and syncope [8]. Furthermore, many patients with chronic conditions who are prescribed more antihypertensives have immense disease costs, which impacts their quality of life; consequently, nonadherence and nonpersistence are common [2, 9–14]. There is consistent evidence that patients under medication could benefit from regular physical exercise when adequately prescribed and consistently practiced. In this case, exercise can act as adjuvant therapy, exposing the patient to both medication and daily exercise [15].

Baduanjin is a mind-body integration of physical activity based on the traditional Chinese medicine theory, which cultivates qi to enhance the body's vitality. It is the most widely practiced form of traditional Chinese Qigong, dating back nearly 2500 years [16]. Baduanjin, also named eight-section brocades because it routinely consists of eight individual movements, is characterized by gentle postures, a meditative mind state, and breathing control, and each movement may also have salutary effects on different parts of the body or specific organs [17]. Recently, several studies have found that Baduanjin has beneficial effects on controlling BP in elderly patients who are intolerant to intense physical activities [18, 19]. Many clinical studies have described that Baduanjin exercise has beneficial effects on stabilizing glucose and lipids and improving quality of life [20–22]. Moreover, a previous randomized controlled trial has shown that short-term endurance exercise has effects on the gut microbiota diversity and composition in elderly individuals [23]. Given these findings, Baduanjin exercise may be suitable for older patients with limited daily activity and long-term antihypertensive treatment. Thus, Baduanjin is expected to become an adjuvant therapy for medication reduction in older patients with HTN.

No previous trial has evaluated the use of Baduanjin exercise-based training as an adjuvant therapy to reduce antihypertensive medications in older patients. Therefore, we designed a single-center, open, randomized controlled trial (RCT) to investigate the effect of Baduanjin exercise acting as adjuvant therapy on antihypertensive medication reduction in older patients with HTN and investigate the changes of office blood pressure, 24-h ambulatory blood pressure, health-related quality of life, and composition of gut microbiota.

## 2. Methods

### 2.1. Study Design

**Figure 1:**
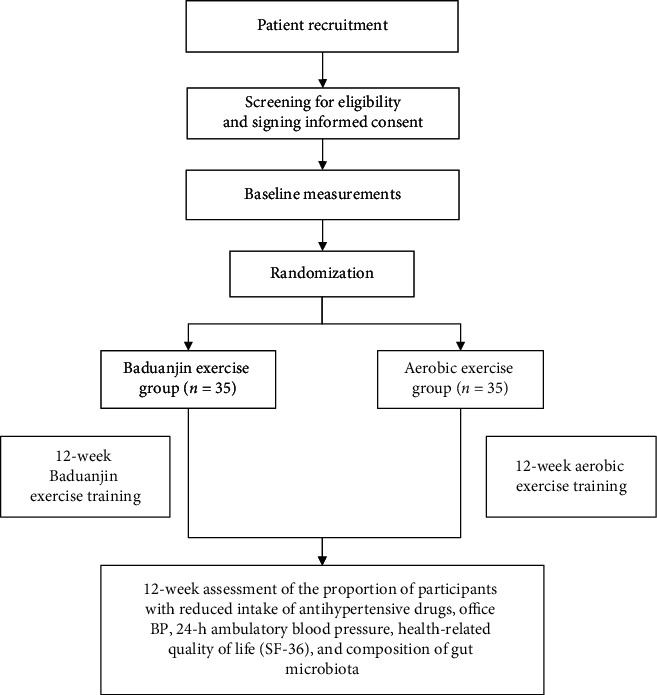
Study flow chart.

This study is a single-center, open, randomized controlled trial that will be conducted at the Guang'anmen Hospital of the Chinese Academy of Medical Sciences. The research period will cover June 2019 to June 2022. Seventy eligible patients will be randomly assigned to either the Baduanjin exercise or aerobic exercise groups for 12 weeks. The outcomes will be assessed at 12 weeks (immediately after the intervention). The study design process is shown in the study flow chart in Figure 1. The protocol of this clinical trial complies with Standard Protocol Items: Recommendations for Interventional Trials guidelines [24] and follows the Consolidated Standards of Reporting Trials [25].

### 2.2. Recruitment

We will adopt a series of recruitment strategies, including recruiting general outpatients and inpatients of the Guang'anmen Hospital of the China Academy of Chinese Medical Sciences Comprehensive Geriatric Department and posting posters in the hospital. The study investigators will communicate with potential patients and discuss the details of the study. The potential participants will be given information about the study, including the study purpose, process, intervention methods (Baduanjin exercise and aerobic exercise), and the possible side effects. A screening procedure will be performed, and those who are eligible and wish to participate will sign informed consent.

### 2.3. Screening Procedure and Eligibility Criteria

Following provision of informed consent, the demographic characteristics, history of disease, medication use (including the name, usage, dosage, and frequency of medications), and office BP will be collected to identify eligible patients. Patients will be eligible to participate in the study if they fit the eligibility criteria. The screening procedure will be completed before randomization.

#### 2.3.1. Inclusion Criteria

1. Male or female, between 65 and 80 years old.
2. Office BP: 120 ≤ systolic pressure (SBP) < 140 mmHg, diastolic pressure <90 mmHg (according to the baseline measured office BP).
3. Stable dose of antihypertensive drugs for at least 3 months.
4. Treated with a moderate dose of two or more antihypertensive drugs for at least 12 months. According to the guidelines, antihypertensive drugs are defined as five major drugs: angiotensin-converting enzyme inhibitors, angiotensin II receptor blockers, beta-blockers, calcium channel blockers, and diuretics (thiazides and thiazide-like diuretics such as chlorthalidone and indapamide).
5. Eligible patients must be willing to participate in the study and provide informed consent.

#### 2.3.2. Exclusion Criteria

1. Secondary HTN or previous accelerated or malignant HTN.
2. Severe cardiovascular diseases, pulmonary diseases, liver diseases, kidney diseases, nervous diseases, psychiatric disorders, cancer, infectious diseases, or others, limiting the ability to participate in the Baduanjin or aerobic exercise programs and influencing the reduction of antihypertensive medications.
3. Using other drugs that may affect BP.
4. Smoking and/or alcohol abuse.
5. Women who are pregnant or planning to become pregnant.
6. Enrolled in other clinical trials in the last 3 months.
7. Practiced Baduanjin or other similar physical activities in the past 3 months, such as Qigong, yoga, or Tai Chi.

### 2.4. Randomization

Each participant will be randomly assigned to either the Baduanjin exercise group or the aerobic exercise group at a ratio of 1 : 1. The allocation sequence will be generated by the SAS software v.9.4 PLAN program and managed by an independent statistician who will not anticipate in the recruitment, evaluation, and intervention of participants. The statistician will put the list in a sealed, opaque, sequentially numbered envelope and give it to the investigators after sealing. Participants will be recruited in a natural, unpredictable order. After screening, the investigator will open an envelope sequentially to obtain a random number and determine whether the participant will be exposed to Baduanjin exercise or not.

### 2.5. Blinding

Baduanjin exercise is a familiar exercise for the Chinese population, and blinding is impossible to achieve at the level of related investigators, exercise coaches, and participants. However, the outcomes will be independently assessed by two statisticians in a blinded manner.

### 2.6. Intervention

#### 2.6.1. Baduanjin Exercise Group

**Figure 2:**
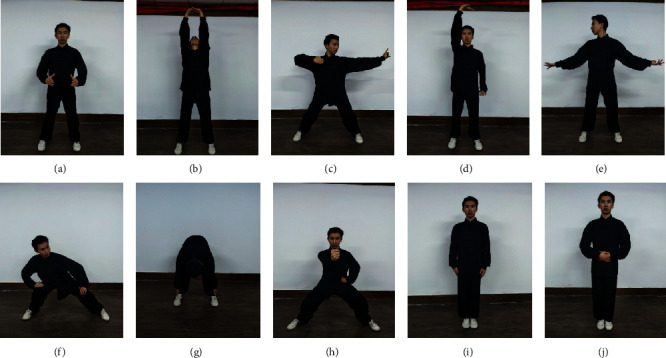
Ten postures of Baduanjin exercise. (a) Preparation posture, (b) Prop up the sky by two hands to improve tri-jiao, (c) Draw a bow on both sides like shooting a vulture, (d) Raise single arm up to regulate spleen (Pi) and stomach (Wei), (e) Look back to treat five strains and seven impairments, (f) Shake the head and wag to expel heart (Xin)-fire, (g) Pull toes with both hands to reinforce the kidney, (h) Clench one's first and glare to increase strength, (i) Rise and fall on tiptoe seven times to treat all diseases, (j) Ending posture.

Participants randomized to the Baduanjin exercise group will collectively practice Baduanjin at the Guang'anmen Hospital of the China Academy of Chinese Medical Sciences. A professional coach at the Beijing University of Traditional Chinese Medicine will guide the training of participants and supervise them throughout the entire intervention period. Each session will last for 30 min (including 5 min of warm-up, 20 min of Baduanjin training, and 5 min of relaxation), 5 times a week, over the course of 12 weeks. The training program will be formulated following the Healthy Qigong Baduanjin published by the State Sports General Administration and will consist of 10 postures in total (Figure 2) (including the preparation pose and the ending pose). In the continuous training, the patient's heart rate (HR) will be monitored by a polar heart rate monitor, and the exercise intensity and energy expenditure will be constant at 55%–75% of the peak HR. All participants will be instructed to maintain their usual activities and refrain from new strength training.

#### 2.6.2. Control Group

Participants randomized to the aerobic exercise group will collectively practice moderate-intensity walking exercise at the Guang'anmen Hospital of the Chinese Academy of Chinese Medical Sciences, at a frequency of 5 times a week, over a 12-week period. A professional coach will be hired to guide the training and supervise the participants throughout the intervention. Each session will consist of a 5-min warm-up, 20-min walking training, and 5-min relaxation. In the continuous training, the patient's HR will be monitored by the polar heart rate monitor, and the exercise intensity and energy expenditure will be constant at 55%–75% of the peak HR. All participants will be instructed to maintain their usual activities and refrain from new strength training.

### 2.7. Outcome Measurements

Table 1 outlines the primary and secondary outcomes to be undertaken during the study. The primary outcome will be examined at postintervention (week 12). The secondary outcomes will include office BP, 24-h ambulatory blood pressure monitoring, health-related quality of life, and gut microbiota composition, all of which will be measured at baseline (week 0) and postintervention (week 12).

#### 2.7.1. Primary Outcome

The primary outcome will be the proportion of participants with antihypertensive medication reduction postintervention; this will be defined as a reduction of at least 15% of total antihypertensives compared with the baseline medical prescription after the 12-week exercise procedure (e.g., patients using 2 or 3 medications need to reduce ≥50% of 1 medication to achieve the primary outcome and patients using 4 or 5 medications need to reduce ≥1 medication). Guidelines recommend that in older patients (>65 years), treated SBP should not be targeted to <120 mmHg; when the SBP is <120 mmHg, the risk of harm appears to increase and outweigh the benefits [4]. Hence, once participants begin to practice Baduanjin or aerobic exercise, they will be required to return for a routine clinical examination every 4 weeks (including office BP measurement and maintenance of antihypertensive drugs); when office BP is <120 mmHg, we will reduce the number of medications taken by the patients.

**Figure 3:**
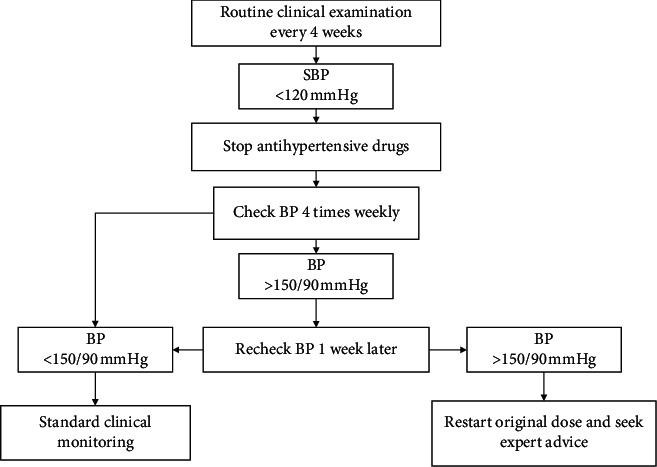
Postexercise medicine reduction monitoring flow chart.

Once a medication has been reduced, research assistants will closely monitor the participant's response to the reduction (through BP). The research assistants will perform the monitoring according to the procedures outlined in Figure 3, although these will be flexible. The participants will be required to return for at least one routine BP check (every 4 weeks); when the patients' BP is >150 mmHg or when adverse events (AEs) occur, the general practitioners (GPs) will be contacted, and the medication (dose or type) will be readjusted to ensure that the incidence of severe AEs occurring by medication will be very low.

*(1) Self-Monitoring*. Participants will optionally self-monitor their BP at home when they reduce their medication. The protocols developed in the Telemonitoring and Self-Management in Hypertension (TASMIN) trials [26, 27] have been modified and simplified to suit this study. These reduced medications will be monitored using revised protocols. Following training, participants will be advised to conduct self-monitoring at least four times during the BP check week (4 times weekly) after drug withdrawal.

If four or more BP readings recorded during the BP check week are higher than clinically safe (e.g., home SBP > 150 mmHg), the research assistants and GP will be contacted, and the medication will be changed according to the original plan. Previous meta-analyses have provided good evidence that self-monitoring differences will not impact the study results but only affect when combined with interventions [28].

#### 2.7.2. Secondary Outcomes

*(1) Office Blood Pressure*. Office BP will be measured using the clinically validated OMRON HBP-1300 medical upper arm sphygmomanometer. Before starting the BP measurement, participants should be relaxed and seated comfortably in a quiet place without speaking for 5 min. An appropriate cuff will be used to measure BP in both arms to detect differences between arms, and the higher value will be used as a reference. Three BP measurements should be recorded at intervals of 1-2 min, and the BP will be recorded as the mean value of the last two readings [29]. For all participants, BP will be measured 1 min and 3 min after standing from a seated position at the first measurement to exclude orthostatic hypotension.

*(2) 24-Hour Ambulatory BP Assessment*. The 24-h ambulatory BP of participants will be collected using a Welch Allyn ambulatory BP monitor 6100 (Skaneateles, NY, USA). During ambulatory BP monitoring, maintaining normal living or working status and avoiding strenuous exercise prevents cuff movement or loosening. If the cuff position moves or is loose, it should be immediately corrected. The measurement interval will be every 30 min during the daytime (6:00 a.m. to 10:00 p.m.) and every 1 h at night (10:00 p.m. to 6:00 a.m.) [30]. The 24-h ambulatory BP monitoring will involve measuring the mean day- and night-time systolic and diastolic 24-h ambulatory BP, coefficient of variation, and average real variability analysis.

*(3) Health-Related Quality of Life*. According to the World Health Organization (WHO), quality of life is defined as an individual's perception of their position in life in the context of the culture and value systems in which they live, and in relation to their goals, expectations, standards, and concerns [31]. This will be assessed according to the Chinese version of the Short Form Health Survey (Short-Form 36). In previous studies, this scale has demonstrated good internal consistency, test-retest reliability, and discriminant validity [32–34]. The scale consists of eight dimensions: physical functioning, role physical, bodily pain, general health, vitality, social functioning, role emotional, and mental health, and two summary components: physical and mental component subscales, ranging from 0 to 100, with higher scores indicating better quality of life [35].

*(4) Composition of Gut Microbiota*. Fecal samples will be collected for subsequent examination, and microbial composition will be analyzed via high-throughput 16S rRNA gene sequencing. We will advise participants not to receive any antibiotics for at least 1 month before sample collection and not consume any food containing probiotics (e.g., yogurt) for 7 days before sample collection.

The 24-h collection kit includes four gel ice packs (Techni Ice), collection bags, and fecal collection instructions and will be provided for fecal sample collection. Research assistants will also give participants detailed oral instructions before they collect fecal samples. In our laboratory, the cool bag will be maintained at 4°C, which has no significant effect on fecal microbiota diversity or composition [31]. Each sample will be stored briefly in a personal collection kit at 4°C and transported to the Guang'anmen Hospital within 24 h. Once at the hospital, the samples will be taken to the laboratory, where they will be aliquoted and stored at −80°C for subsequent analysis of intestinal microbial composition. All microbial community genomic DNA extractions will be performed using the E.Z.N.A.® Soil DNA Kit (Omega Bio-tek, Norcross, GA, USA).

The hypervariable region V3-V4 of the bacterial 16S rRNA gene will be amplified with primer pairs 338F and 806R using an ABI GeneAmp® 9700 PCR thermocycler (ABI, CA, USA) to obtain a polymerase chain reaction (PCR) product. The PCR product will then be purified and quantified, and the library will be constructed. High-throughput sequencing will be performed using the Illumina MiSeq platform (Illumina, San Diego, USA). The raw 16S rRNA gene sequencing reads will be demultiplexed, quality-filtered using Trimmomatic, and merged by FLASH. Operational taxonomic units (OTUs) will be clustered using UPARSE (version 7.1, http://drive5.com/uparse/), and the Quantitative Insights Into Microbial Ecology (QIIME) software will be used to analyze changes in relative bacterial abundance and *α* and *ß* diversity.

### 2.8. Sample Size

We hypothesize that at least a 30% difference in this primary outcome will be detected after the 12-week exercise intervention (defined as a reduction of at least 15% of the drug prescription for each patient) [36]. This 30% absolute difference represents the difference between the assumed 40% proportion of outcome incidence in the Baduanjin exercise group and 10% incidence in the aerobic exercise group. Assuming a two-sided significance level of 5% and a power of 90%, a sample size of 60 patients (30 in each group) will be needed. Considering sample dropout, increasing the sample size by 10%, a total of 68 cases will need to be studied in the two groups. Thus, the sample size was expanded to include 70 cases (35 cases in each group).

### 2.9. Data Collection and Management

After recruiting participants, investigators and a professional coach will guide the training of participants and supervise them throughout the entire intervention period. Especially, participants will be required to return for a routine clinical examination every 4 weeks and monitor the participant's response through WeChat until the end of the study. As for the participants who discontinue or deviate from intervention protocols, investigators will call and WeChat participants to minimize data loss and participant withdrawal or loss during the study period.

Every participant should follow the instruction during the study period, and their related information should be recorded and stored as a print-based case report form (p-CRF). The electrical CRF should be stored in an encrypted mobile hard disk along with the procedure of filling in the p-CRF. All basic information of participants will be protected by the researching group, and a unique identified number will be applied during the procedure of the study. The mobile hard disk, p-CRF, and informed consent will be stored in the office of the Guang'anmen Hospital of the China Academy of Chinese Medical Sciences.

### 2.10. Data Monitoring

An independent Data and Safety Monitoring Board (DSMB) comprising two cardiologists and a statistician will monthly safeguard trial participants, monitor emerging trial data (including identifying any trends, such as increases in unexpected events), and pause the trial when necessary. The statistician of DSMB will also compare the data between the p-CRF and electrical CRF, and any modification of the data will be monitored and recorded in detail.

### 2.11. Statistical Analysis

This study will be based on intention to treat (ITT), and the results will be analyzed in accordance with the principle of ITT analysis. Statistical analysis will be performed at two prespecified time points and will be used to determine primary and secondary outcomes. Data from all patients who complete the follow-up will be analyzed according to the group in which they were originally defined. Participants who received reduced medication in the primary outcome analysis will be defined as those who received reduced medication during the exercise intervention and maintained it until the end of the study. The chi-square test or Fisher's exact test will be used to compare the proportion of patients with a primary outcome event between the two study groups. For the secondary outcomes, measurement data will be expressed as mean ± standard deviation (SD), and independence test, normality test, and homogeneity test of variance will be performed for each group. In addition, repeated measurements over time will be analyzed using analysis of variance (ANOVA of repeated measures), and pair comparisons between groups will be performed using the LSD *t*-test (homogeneity of variance) or Tamhane's T2 test (heterogeneity of variance). The composition of the gut microbiota will be analyzed using a nonparametric Mann–Whitney U test for relative bacterial abundance (at baseline and between groups). A Wilcoxon matched-pairs test will be used for comparison to analyze the differences in relative bacterial abundance between the Baduanjin and aerobic intervention groups. A *P*

-value of <0.05 will be taken to indicate a significant difference. All analyses will be performed using the SPSS v.17.0 software package. Every effort will be made to minimize data loss and participant withdrawal or loss during the study period; if the primary outcome or potential confounders' data are missing, a multiple imputation method will be used to fill in missing data.

### 2.12. Safety Assessment

AEs may include medication withdrawal AEs (defined as the 10 most commonly reported AEs: stiff joints, pain, fatigue, loss of strength, breathlessness, sleep difficulties, pins and needles, sore eyes, dizziness, and impotence), exercise injury, and fall [27]. Serious AEs (SAEs) include, among others, prolonged hospital stay, disability, and death. AEs and SAEs will be recorded during the 12 weeks of intervention from enrollment. If either occurs during the study, the investigators will evaluate the relationship between the events and the intervention and report it to the Ethical Committee and DSMB; subsequently, they will decide whether the participant should withdraw from the trial.

## 3. Discussion

We will investigate whether Baduanjin exercise could be used as an adjuvant therapy for medication reduction in older patients with HTN and whether it has a positive effect on the quality of life. High BP has been found to be associated with lower gut microbiota alpha diversity in prior studies [37–39]. In addition, microbiota composition, including lower short-chain fatty acids (SCFA)-producing bacteria and higher Gram-negative species, is specific differences between hypertensive and normotensive patients [40–44]. A previous randomized controlled trial demonstrated that exercise training increases fecal SCFA concentrations and gut microbiome SCFA-producing capacity and influences BP by interacting with host SCFA receptors [45, 46]. Baduanjin may be continuously and positively associated with gut microbiota composition. We will also explore its mechanism of influence on BP based on the composition of gut microbiota. If successful, this trial will provide patients with HTN with a good exercise program and be encouraged as an adjuvant therapy to reduce the amount of medications required by patients.

As a traditional exercise in China, Baduanjin has been used extensively in medical care because of its functions in regulating organs and dredging meridians [32]. Baduanjin exercise is gentle and straightforward and is particularly suitable for elderly patients with HTN who have reduced mobility. Baduanjin exercise is easy to master and may improve poor adherence and nonpersistence in taking antihypertensive agents [33]. Therefore, if it is proven to be effective, Baduanjin could be a useful adjuvant therapy to reduce the number of drugs in elderly patients with HTN.

The trial will use a rigorous random allocation method, blinded to evaluators and statistical analysts, to reduce bias. Therefore, the test is expected to produce reliable results. However, there are some potential limitations to this test. First, although the sample size has been calculated according to the existing literature and standard formula, the sample size is small and not sufficiently representative. Second, it is challenging to monitor other physical activities of the participants during the study. Although all participants will be required to return to the hospital regularly for supervised intervention, this will not be sufficient to ensure that participants do not exercise outside the intervention period. Finally, due to the nature of the exercise intervention, it is not possible to blind participants, coaches, and investigators in the experiment. However, every effort will be made to ensure that the outcome evaluators and statistical analysts are not aware of allocation of the treatment options.

In conclusion, this trial will evaluate Baduanjin as an adjuvant therapy for medication reduction in older individuals with HTN.

The results of this RCT will help provide evidence to verify the effects of Baduanjin as an adjuvant therapy for reducing the demand for antihypertensives to find the best treatment for controlling HTN.

## Figures and Tables

**Table 1:** Schedule of trial measurements for primary and secondary outcomes.

Study phase		Screen/enroll	Allocation	Intervention period	Intervention end
Time points		Week 0		Week 12	
Screening/enrollment	Eligibility screening	●	●	—	—
	Informed consent	●	—	—	—
	Baseline measurement	●	—	—	—
	Random allocation	—	●	—	—

Intervention	Baduanjin exercise	—	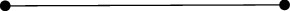		
	Aerobic exercise (walking)	—	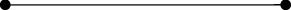		

Outcome assessment	Proportion of participants with reduced intake of antihypertensive drugs	—	—	—	●
	Office blood pressure	●	●	—	●
	24-hour ambulatory blood pressure	—	●	—	●
	Health-related quality of life (SF-36)	—	●	—	●
	Composition of gut microbiota	—	●	—	●

Adverse events	—	—	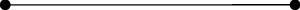		

## Data Availability

The data used to support the study are available from the corresponding author upon request.

## Abbreviations

HTN: Hypertension
ESC: European Society of Cardiology
ESH: European Society of Hypertension
BP: Blood pressure
RCT: Randomized controlled trial
SBP: Systolic pressure
HR: Heart rate
AE: Adverse event
GP: General practitioner
TASMIN: Telemonitoring and Self-Management in Hypertension
WHO: World Health Organization
PF: Physical functioning
RP: Role physical
OUT: Operational taxonomic unit
QIIME: Quantitative Insights Into Microbial Ecology
CRF: Case report form
DSMB: Data and Safety Monitoring Board
ITT: Intention to treat
SD: Standard deviation
SCFA: Short-chain fatty acids.

## References

[1] Lawes C. M., Vander Hoorn S., Rodgers A., International Society of Hypertension (2008). Global burden of blood-pressure-related disease, 2001. *Lancet*.

[2] Wang J.-G. (2018). Unique approaches to hypertension control in China. *Annals of Translational Medicine*.

[3] Lacey B., Lewington S., Clarke R. (2018). Age-specific association between blood pressure and vascular and non-vascular chronic diseases in 0.5 million adults in China: a prospective cohort study. *Lancet Global Health*.

[4] ESC/ESH (2019). 2018 ESC/ESH guidelines for the management of arterial hypertension: Erratum. *Journal of Hypertension*.

[5] Lu J., Lu Y., Wang X. (2017). Prevalence, awareness, treatment, and control of hypertension in China: data from 1·7 million adults in a population-based screening study (China PEACE Million Persons Project). *The Lancet*.

[6] Bejan-Angoulvant T., Saadatian-Elahi M., Wright J. M. (2010). Treatment of hypertension in patients 80 years and older: the lower the better? A meta-analysis of randomized controlled trials. *Journal of Hypertension*.

[7] Benetos A., Labat C., Rossignol P. (2015). Treatment with multiple blood pressure medications, achieved blood pressure, and mortality in older nursing home residents. *JAMA Internal Medicine*.

[8] Sink K. M., Evans G. W., Shorr R. I. (2018). Syncope, hypotension, and falls in the treatment of hypertension: results from the randomized clinical systolic blood pressure intervention trial. *Journal of the American Geriatrics Society*.

[9] Dahlöf B., Sever P. S., Poulter N. R. (2005). Prevention of cardiovascular events with an antihypertensive regimen of amlodipine adding perindopril as required versus atenolol adding bendroflumethiazide as required, in the Anglo-Scandinavian Cardiac Outcomes Trial-Blood Pressure Lowering Arm (ASCOT-BPLA): a multicentre randomised controlled trial. *The Lancet*.

[10] Wright J. M., Musini V. M., Gill R. (2018). First-line drugs for hypertension. *The Cochrane Database of Systematic Reviews*.

[11] Wang C., Lang J., Xuan L., Li X., Zhang L. (2017). The effect of health literacy and self-management efficacy on the health-related quality of life of hypertensive patients in a western rural area of China: a cross-sectional study. *International Journal for Equity in Health*.

[12] Chowdhury R., Khan H., Heydon E. (2013). Adherence to cardiovascular therapy: a meta-analysis of prevalence and clinical consequences. *European Heart Journal*.

[13] Panjabi S., Lacey M., Bancroft T., Cao F. (2013). Treatment adherence, clinical outcomes, and economics of triple-drug therapy in hypertensive patients. *Journal of the American Society of Hypertension*.

[14] Abegaz T. M., Shehab A., Gebreyohannes E. A., Bhagavathula A. S., Elnour A. A. (2017). Nonadherence to antihypertensive drugs. *Medicine*.

[15] Moraes-Silva I. C., Mostarda C. T., Silva-Filho A. C., Irigoyen M. C. (2017). Hypertension and exercise training: evidence from clinical studies. *Advances in Experimental Medicine and Biology*.

[16] Huang J., Wang X. Y. (2012). [Review of centennial development of Qigong]. *Zhonghua Yi Shi Za Zhi*.

[17] Koh T. C. (1982). Baduanjin—an ancient Chinese exercise. *The American Journal of Chinese Medicine*.

[18] Wang J., Xiong X. (2013). Evidence-based Chinese medicine for hypertension. *Evidence-Based Complementary and Alternative Medicine*.

[19] Xiong X., Wang P., Li S., Zhang Y., Li X. (2015). Effect of Baduanjin exercise for hypertension: a systematic review and meta-analysis of randomized controlled trials. *Maturitas*.

[20] Mei L., Chen Q., Ge L., Zheng G., Chen J. (2012). Systematic review of Chinese traditional exercise Baduanjin modulating the blood lipid metabolism. *Evidence-Based Complementary and Alternative Medicine*.

[21] An T., He Z. C., Zhang X. Q. (2019). Baduanjin exerts anti-diabetic and anti-depression effects by regulating the expression of mRNA, lncRNA, and circRNA. *Chinese Medicine*.

[22] Bao X., Qiu Q.-X., Shao Y.-J., Quiben M., Liu H. (2020). Effect of sitting Ba-Duan-Jin exercises on balance and quality of life among older adults: a preliminary study. *Rehabilitation Nursing*.

[23] Taniguchi H., Tanisawa K., Sun X. (2018). Effects of short‐term endurance exercise on gut microbiota in elderly men. *Physiological Reports*.

[24] Chan A.-W., Tetzlaff J. M., Altman D. G. (2013). SPIRIT 2013 statement: defining standard protocol items for clinical trials. *Annals of Internal Medicine*.

[25] Schulz K. F., Altman D. G., Moher D. (2010). CONSORT 2010 statement: updated guidelines for reporting parallel group randomized trials. *Annals of Internal Medicine*.

[26] McManus R. J., Mant J., Haque M. S. (2014). Effect of self-monitoring and medication self-titration on systolic blood pressure in hypertensive patients at high risk of cardiovascular disease. *JAMA*.

[27] Sheppard J. P., Burt J., Lown M. (2020). Effect of antihypertensive medication reduction vs. usual care on short-term blood pressure control in patients with hypertension aged 80 years and older. *JAMA*.

[28] Tucker K. L., Sheppard J. P., Stevens R. (2017). Self-monitoring of blood pressure in hypertension: a systematic review and individual patient data meta-analysis. *PLOS Medicine*.

[29] Unger T., Borghi C., Charchar F. (2020). 2020 international society of hypertension global hypertension practice guidelines. *Hypertension*.

[30] Muntner P., Shimbo D., Carey R. M. (2019). Measurement of blood pressure in humans: a scientific statement from the American heart association. *Hypertension (Dallas, Tex.: 1979)*.

[31] WHO (1995). The world health organization quality of life assessment (WHOQOL): position paper from the world health organization. *Social Science & Medicine*.

[32] Wang S., Fan W., Yu W. (2016). [Analysis on reliability and validity of SF-36 scale in urban residents]. *Zhonghua Liu Xing Bing Xue Za Zhi*.

[33] Bunevicius A. (2017). Reliability and validity of the SF-36 Health Survey Questionnaire in patients with brain tumors: a cross-sectional study. *Health and Quality of Life Outcomes*.

[34] Lin Y., Yu Y., Zeng J., Zhao X., Wan C. (2020). Comparing the reliability and validity of the SF-36 and SF-12 in measuring quality of life among adolescents in China: a large sample cross-sectional study. *Health and Quality of Life Outcomes*.

[35] Alonso J., Prieto L., Anto J. M. (1995). [The Spanish version of the SF-36 Health Survey (the SF-36 health questionnaire): an instrument for measuring clinical results]. *Medicina Clínica (Barc)*.

[36] Schiavon C. A., Bersch-Ferreira A. C., Santucci E. V. (2018). Effects of bariatric surgery in obese patients with hypertension. *Circulation*.

[37] Yang T., Santisteban M. M., Rodriguez V. (2015). Gut dysbiosis is linked to hypertension. *Hypertension*.

[38] De La Cuesta-Zuluaga J., Mueller N. T., Alvarez-Quintero R. (2019). Higher fecal short-chain fatty acid levels are associated with gut microbiome dysbiosis, obesity, hypertension and cardiometabolic disease risk factors. *Nutrients*.

[39] Jackson M. A., Verdi S., Maxan M.-E. (2018). Gut microbiota associations with common diseases and prescription medications in a population-based cohort. *Nature Communications*.

[40] Dan X., Mushi Z., Baili W. (2019). Differential analysis of hypertension-associated intestinal microbiota. *International Journal of Medical Sciences*.

[41] Huart J., Leenders J., Taminiau B. (2019). Gut microbiota and fecal levels of short-chain fatty acids differ upon 24-hour blood pressure levels in men. *Hypertension*.

[42] Li J., Zhao F., Wang Y. (2017). Gut microbiota dysbiosis contributes to the development of hypertension. *Microbiome*.

[43] Sun S., Lulla A., Sioda M. (2019). Gut microbiota composition and blood pressure. *Hypertension*.

[44] Verhaar B. J. H., Collard D., Prodan A. (2020). Associations between gut microbiota, faecal short-chain fatty acids, and blood pressure across ethnic groups: the HELIUS study. *European Heart Journal*.

[45] Allen J. M., Mailing L. J., Niemiro G. M. (2018). Exercise alters gut microbiota composition and function in lean and obese humans. *Medicine & Science in Sports & Exercise*.

[46] Pluznick J. L. (2017). Microbial short-chain fatty acids and blood pressure regulation. *Current Hypertension Reports*.

